# Enteric Permeability, Systemic Inflammation, and Post-Discharge Growth Among a Cohort of Hospitalized Children in Kenya and Pakistan

**DOI:** 10.1097/MPG.0000000000003619

**Published:** 2022-09-20

**Authors:** Kirkby D. Tickell, Donna M. Denno, Ali Saleem, Asad Ali, Zaubina Kazi, Benson O. Singa, Catherine Otieno, Charles Mutinda, Victor Ochuodho, Barbra A. Richardson, Kristjana H. Ásbjörnsdóttir, Stephen E. Hawes, James A. Berkley, Judd L. Walson

**Affiliations:** From the * Department of Global Health, University of Washington, Seattle, Seattle, WA; † The Childhood Acute Illness & Nutrition (CHAIN) Network, Nairobi, Kenya; the ‡ Department of Pediatrics, University of Washington, Seattle, Seattle, WA; the § Department of Pediatrics, Aga Khan University, Karachi, Pakistan; the ‖ Centre for Clinical Research, Kenya Medical Research Institute, Nairobi, Kenya; the ¶ Department of Biostatistics, University of Washington, Seattle, Seattle, WA; the # Department of Epidemiology, University of Washington, Seattle, Seattle, WA; the ** School of Health Sciences, University of Iceland, Reykjavik, Iceland; the †† KEMRI/Wellcome Trust Collaborative Research Programme, Kilifi, Kenya; the ‡‡ Nuffield Department of Medicine, University of Oxford, Oxford, United Kingdom; the §§ Department of Medicine, University of Washington, Seattle, Seattle, WA.

**Keywords:** childhood growth, environmental enteric function, systemic inflammation

## Abstract

To determine whether gut permeability is associated with post-discharge growth and systemic inflammation among hospitalized children in low- and middle-income countries.

**Methods:**

Children aged 2–23 months being discharged from Civil Hospital Karachi (Pakistan) and Migori County Referral Hospital (Kenya) underwent lactulose-rhamnose ratio (LRR) permeability testing and were compared to age-matched children from their home communities. Linear mixed effect models estimated the associations between LRR among discharged children with change in length-for-age (LAZ) and weight-for-age *z* score (WAZ) at 45, 90, and 180 days after discharge. Linear regression tested if relationships between LRR, systemic inflammation [C-reative protein (CRP), Cluster of Differentiation 14 (CD14), Tumour Necrosis Factor Alpha (TNFα), Interleukin-6 (IL-6)], and enterocyte damage [Intestinal Fatty-Acid Binding protein (I-FABP)] differed between the hospitalized and community groups.

**Results:**

One hundred thirty-seven hospitalized and 84 community participants were included. The hospitalized group had higher log-LRR [0.43, 95% confidence interval (CI): 0.15–0.71, *P* = 0.003] than the community children. Adjustment for weight-for-length *z* score at discharge attenuated this association (0.31, 95% CI: 0.00–0.62, *P* = 0.049). LRR was not associated with changes in WAZ or LAZ in the post-discharge period. Associations between LRR and CRP (interaction *P* = 0.036), TNFα (*P* = 0.017), CD14 (*P* = 0.078), and IL-6 (*P* = 0.243) differed between community and hospitalized groups. LRR was associated with TNFα (*P* = 0.004) and approached significance with CD14 (*P* = 0.078) and IL-6 (*P* = 0.062) in community children, but there was no evidence of these associations among hospitalized children.

**Conclusions:**

Although increased enteric permeability is more prevalent among children being discharged from hospital compared to children in the community, it does not appear to be an important determinant of systemic inflammation or post-discharge growth among hospitalized children.

**What Is Known**

- Enteropathy is an important determinant of child growth and health among relatively healthy children in Africa and Asia.
- Systemic inflammation has been shown to be one of the important mediators in the link between enteropathy and growth in community cohort studies.

**What Is New**

- Enteropathy, measured by enteric permeability, was higher among hospitalized children compared to similar children in the community.
- Enteropathy was not linked to subsequent growth or systemic inflammation among hospitalized children.
- Enteropathy may not be an important determinant of recovery among acutely unwell children.

Children discharged from hospitals in sub-Saharan Africa and Asia remain at high risk of poor health outcomes for the subsequent 6–12 months (1–3). Poor growth is also common in the post-discharge period (4,5), and has been linked to life-long morbidities, including impaired cognitive development and noncommunicable diseases (6,7). Enteropathies are increasingly understood to be highly prevalent determinants of child growth in sub-Saharan Africa and Asia (7–10). They may be caused by exposure to a contaminated environment (environmental enteric dysfunction), or diseases such as Human Immunodeficiency Virus (HIV), kwashiorkor, zinc deficiency, celiac disease, or inflammatory bowel disease (9,11,12). Risk factors for enteropathy are highly prevalent among hospitalized children, suggesting that gut dysfunction may be common among these children.

Dual sugar testing, in which the ratio of a large sugar that should not cross a normal gut barrier (eg, lactulose) to a small sugar which readily crosses (eg, rhamnose), can assess enteric barrier function (13). Dual sugar testing studies have demonstrated a high prevalence of childhood enteropathy in sub-Saharan Africa and Asia, and have linked worse enteric barrier function to poor childhood growth. These studies suggest that systemic inflammation may be a key mediator between enteropathy and adverse outcomes (8,14). However, little is known about the role of enteric permeability among children recovering from acute illnesses. If enteric permeability undermines recovery from acute illness, it could be an important interventional target to improve post-discharge health.

We assessed the relationship between gut permeability and subsequent growth among children discharged from hospital in Kenya and Pakistan. We hypothesized that permeability, measured by the lactulose-rhamnose ratio (LRR), would be elevated among discharged children compared to similar aged children from the same community, and that LRR is associated with biomarkers of enterocyte damage and systemic inflammation at discharge and poor post-discharge growth. Finally, we tested whether relationships between LRR and biomarkers of enterocyte damage and systemic inflammation differed between the community and hospitalized groups.

## METHODS

### Parent Study

This sub-study recruited Childhood Acute Illness and Nutrition (CHAIN) cohort participants at the Civil Hospital Karachi (March 2018–September 2019) and the Migori County Referral Hospital (December 2017–October 2019) (15,16). CHAIN aimed to understand the cause of inpatient and post-discharge mortality among children under 2 years of age. Migori Hospital is a district referral facility in rural Kenya. Migori is malaria endemic, one-quarter of its children are stunted [length-for-age *z* score (LAZ) < −2], and the HIV prevalence is among the highest in Kenya (17). Civil Hospital is an urban referral facility in Pakistan. HIV and malaria are rare, but over one-third of children are stunted and the wasting prevalence [weight-for-length *z* score (WLZ) < −2] is 3 times higher than in Migori (18).

Children aged 2–23 months admitted to these hospitals were eligible for CHAIN. Children with a traumatic injury, a condition requiring surgery within 6 months, or those whose parents did not consent were excluded. To oversample children at high risk of mortality, CHAIN stratified enrollment into these groups: (1) severely low mid-upper arm circumference (MUAC, <11.5 cm if >5 months old, otherwise <11 cm) or nutritional edema, (2) moderately low MUAC (≥11.5 cm but <12.5 cm if >5 months, otherwise ≥11 cm but <12.0 cm), and (3) a normal MUAC (≥12.5 cm if >5 months, otherwise ≥12.0 cm). Clinical and anthropometric data were collected at admission and discharge. All staff were trained on anthropometric measurements, length was measured in centimeters on SECA (Chino, CA, USA) 416 length boards, and weight was collected in kilograms using SECA 354 scales with an infant basin or standing scales for older children. Home characteristics were assessed at home visits after discharge. Follow-up was at 45, 90, and 180 days after discharge to record vital status and anthropometric changes.

At home visits, community participants were recruited from households near the index-hospitalized child’s home using pseudo-random selection (third house to the north of the index home). A child without history of acute illness in the last 14 days in the same age bracket as the index-hospitalized child (<6, 6–11, 12–23 months) was recruited. If an eligible child was not identified, the next household continuing north was selected. Community participants underwent the same evaluation as hospitalized children.

### Sub-Study Enrollment

Children in CHAIN hospitalized cohort who became medically stable (no respiratory distress, no supplemental oxygen, taking feeds orally) were eligible for this sub-study. The first 3 eligible children each week were selected for participation to encourage accurate LRR testing. Children with diarrhea, defined by clinicians or caregivers reporting loose stool, on the day of the LRR test were excluded, as lactulose exacerbates diarrhea.

### The Lactulose-Rhamnose Test

Children fasted for 1 hour, and pre-dose urine samples were collected during this time. A 10 mL oral solution containing 1000 mg lactulose and 200 mg L-rhamnose was administered after the fasting hour, urine passed in the next 20 minutes was discarded, then all urine passed during the subsequent 2 hours was collected. Children were encouraged to drink water or breastfeed during the collection phase. Contamination of urine with stool, urine leakage, or failure to void in the collection period were test failures. Failed tests were repeated after 24 hours if caregivers were willing.

Urine from 3 time-periods (pre-dose, 20–80 minutes, and 80–140 minutes post-administration) were aliquoted into cryovials containing 50 µL chlorhexidine and stored at −80°C. High performance liquid chromatography mass spectrometry (Mayo Clinic, Rochester, MN) quantified lactulose and rhamnose recovery, the LRR in each aliquot, and the cumulative post-dose LRR (Supplemental Digital Content 1.0 & 2.1, http://links.lww.com/MPG/C941). Failure to detect rhamnose in post-administration samples was considered a test failure. Lactulose concentrations below the limit of detection in post-dose samples were replaced with the lower limit of detection divided by the square root of 2 (19).

### Plasma Biomarkers

Blood collected at discharge was processed within 1 hour of collection and stored at −80°C. CHAIN selected 111 hospitalized and 64 community children from this sub-study for a SomaScan (Somalogic Inc., Boulder CO. USA) proteomic analysis (20,21). Five biomarkers associated with poor growth and intestinal permeability—CD14, CRP, IL-6, TNFα (inflammatory), and I-FABP (enterocyte damage)—from this panel were used to test if LRR had similar associations with these biomarkers in the community and hospitalized groups.

### Statistical Methods

#### Sample Size

The study was powered to detect a difference in LRR between hospitalized and community children. With 130 hospitalized, 80 community children, 80% power, and 0.05 alpha, the minimum detectable difference was ±0.50, if the LRR of community children was 0.4 [1.2 standard deviations (SD)]. For the growth analysis, we would have 80% power (alpha: 0.05) to detect a ±0.11 ΔLAZ difference, when comparing 98 hospitalized children in the lower 3-quarters of LRR values to those in the top-quarter, assuming the 98 children had a ΔLAZ of 0.0 (0.2 SD).

#### Prevalence Comparison

Median and range of LRR, percentage excretions, and pre-sugar administration of urinary lactulose and rhamnose concentrations were calculated for hospitalized and community children. CHAIN oversampled wasted children, therefore each World Health Organization defined wasting category (moderate: WLZ < −2 and ≥−3, and if *>*6 months MUAC < 12.5 cm and ≥11.5 cm; severe: WLZ < −3, edema, and if *>*6 months MUAC <11.5 cm) was also compared to community children using the Wilcoxon rank-sum test. Community children were not disaggregated by wasting status because their recruitment was not skewed toward wasting. LRR scores were also compared to the 95th percentile LRR value derived from children of similar age in the United States (LRR > 0.7) (19). Linear regression of log-transformed LRR was used to adjust for confounding, with site as a random effect. Log-transformed ratios perform better than untransformed ratios in models (13). Confounders were tested in a forward stepwise fashion and retained if the effect estimate changed by greater than 10%. Potential confounders included age (continuous), sex, any current breastfeeding (binary), currently exclusively breastfeeding (binary), WLZ (continuous), LAZ (continuous), weight-for-age *z* score (WAZ; continuous), improved sanitation (binary), and improved water source (binary). A sensitivity analysis excluding HIV infected children was also performed.

#### Post-Discharge Growth

The growth analysis included hospitalized children with LRR results who survived to day 180 without being lost to follow-up. Community children had no follow-up so were not included in this analysis. Length and weight were converted to age-standardized *z* scores (22). Linear mixed effect models with a random effect for patient estimated the association between the log-transformed LRR and change in LAZ (ΔLAZ) and change in WAZ (ΔWAZ) (13). WLZ and MUAC were not assessed. WLZ compounds measurement error and is statistically inferior to WAZ and LAZ (23), and MUAC is highly correlated with WAZ. Missing anthropometric data was assumed to be missing at random and imputed using Markov Chain Monte Carlo models based on site, age, sex, recent diarrhea, timepoint, and baseline anthropometric measurements. Crude models included log-LRR, timepoint (dummy variable), and interaction terms between timepoint and LRR to allow the effect of LRR to differ across timepoints. Adjusted models included a priori identified confounders: age (months), sex, recent diarrhea (reported at admission or observed during hospitalization), LAZ, and WAZ at discharge. Finally, to assess the effect of recent diarrhea, the adjusted model was applied to children with diarrhea, and separately to children without diarrhea. Quadratic LRR and age terms were tested to asses linearity, and found to be nonsignificant. The final model was also replicated using percentage lactulose recovery, an alternative measure of permeability.

#### Plasma Biomarkers

The consistency of associations between LRR and the biomarkers across hospitalized and community groups was assessed through an interaction term between log-LRR and recruitment group (community/hospital) in linear regressions. The biomarker values were standardized by subtracting the biomarker’s mean concentration and dividing by its standard deviation. These models were adjusted for patient age (months), sex, site of recruitment, LAZ, and WAZ. Diarrhea status was not included as the community group excluded children with acute illness in the last 14 days. Final adjusted models were replicated using percentage lactulose.

Analyses were conducted in STATA 14.0. StataCorp, College Station, Texas, USA Ethical approval was obtained from the Aga Khan University, the Kenya Medical Research Institute, the University of Washington, and the University of Oxford. All primary caregivers gave written informed consent.

## RESULTS

**FIGURE 1.**
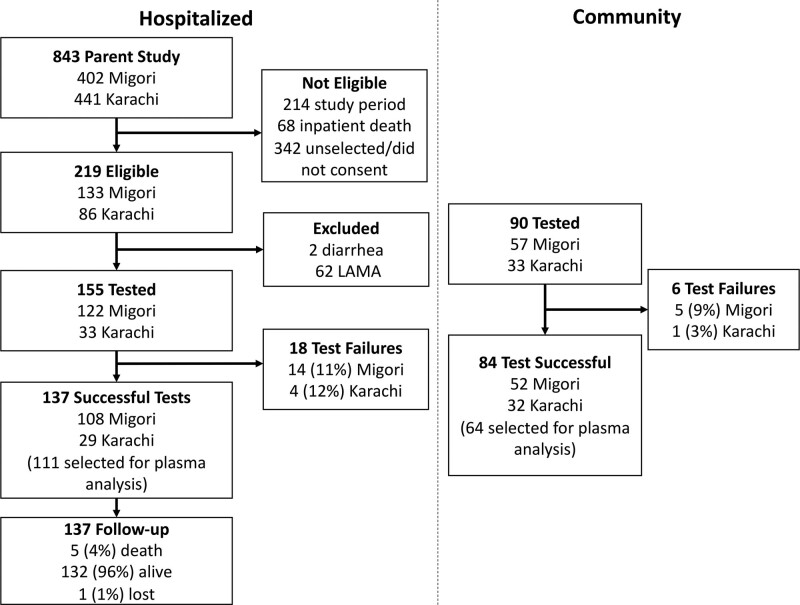
Study flow chart.

At discharge, 155 children were eligible for LRR testing and 137 (88%) tests were successful (Fig. 1).

Test failures were due to undetectable post-dose rhamnose (n: 9), lack of voiding after sugar administration (n: 8), and implausible lactulose (>13 SD above the mean, n: 1). Ninety community children were eligible and 84 (93%) had successful tests; all failed tests were due to a lack of voiding. The hospitalized children’s median age was 9 months [interquartile Range (IQR): 5–14, Table 1]. The median age of community children was also 9 months (IQR: 5–15 months). Fifty-five (40%) hospitalized children had diarrhea at admission or during hospitalization. A description of lactulose and rhamnose recovery and LRR values by site, hospital/community status, and time period is given in Supplemental Digital Content 2.2, http://links.lww.com/MPG/C941.

**TABLE 1.** Participant characteristics

	Hospitalized N = 137	Community N = 84
	n (%)	n (%)
Child		
Migori	108 (79)	52 (62)
Karachi	29 (21)	32 (38)
Age, mo		
<6	39 (28)	20 (24)
6–11	43 (31)	28 (33)
12–23	55 (40)	36 (43)
Sex (male)	90 (66)	49 (58)
Currently breast-feeding	90 (66)	65 (77)
Currently exclusively breastfed	35 (26)	51 (61)
Length of stay		
<48 hrs	25 (18)	-
48 hrs–5 d	57 (42)	-
>5 d	55 (40)	-
Recent antibiotics*	132 (96)	14 (17)
Stunted†	20 (15)	15 (18)
LAZ [mean (SD)]	−1.4 (2.2)	−0.8 (2.3)
Moderately wasted†	31 (23)	9 (11)
Severely wasted†	32 (23)	1 (1)
WLZ [mean (SD)]	−1.4 (1.6)	0.7 (1.5)
WAZ [mean (SD)]	−1.8 (1.8)	−0.4 (1.9)
MUAC [mean (SD)]	12.4 (1.4)	13.8 (1.2)
Discharge diagnosis		
Diarrhea‡	55 (40)	-
Malaria§	29 (21)	1 (1)
LRTI§	64 (47)	-
HIV status		
Unexposed	114 (83)	70 (83)
Exposed, uninfected	16 (12)	13 (15)
Infected	7 (5)	1 (1)
Chronic condition‖	13 (9)	3 (4)
Caregiver		
Biological mother is primary caregiver	126 (92)	79 (94)
Caregiver education¶		
None	17 (12)	16 (19)
Primary	79 (58)	44 (52)
Secondary	35 (26)	22 (26)
Body mass index		
Underweight (<20)	19 (14)	5 (6)
Normal (20–25)	92 (67)	51 (61)
Overweight (>25)	23 (17)	26 (31)
Household		
Livestock ownership¶	80 (58)	47 (56)
Improved water source¶#	93 (68)	60 (71)
Improved sanitation¶#	67 (49)	44 (52)
Food insecurity		
Low	51 (37)	31 (37)
Moderate	57 (42)	42 (50)
High	29 (21)	11 (13)

LAZ = length-for-age *z* score; LRTI = lower respiratory tract infection; MUAC = mid upper arm circumference; SD = standard deviations; WAZ = weight-for-age *z* score; WLZ = weight-for-length *z* score; HIV = Human immunodeficiency Virus.

* In the 7 d before hospital or during the admission.

† Anthropometry at discharge for hospitalized children, enrollment for community. WHO definition of moderate/severe wasting: WHZ < −2, edema, MUAC < 12.5 cm if older than 5 mo.

‡ Not assessed for children in the community.

§ Malaria RDT positive.

‖ Chronic conditions: 5 sickle cell disease (# in Migori hospitalized and # in Migori community cohorts), 11 thalassemia (# in Karachi hospitalized and # in Karachi community cohorts).

¶ Missing data: caregiver education—6 hospitalized, 2 community; livestock—5 hospitalized; water source—6 hospitalized; toilet type—5 hospitalized.

#Improved water and sanitation defined by United Nations definition.

### Community Versus Hospital

Crude LRR was significantly higher among hospitalized (median: 0.36, IQR: 0.22–0.86) compared to community children (median: 0.27, IQR: 0.17–0.46, *P* = 0.013, Table 2). The LRR was higher in hospitalized children in all 3 nutritional strata compared to the community children. Severely wasted hospitalized children had a median LRR of 0.40 (IQR: 0.28–1.01, *P* = 0.023), those with moderate wasting had a median of 0.34 (IQR: 0.22–1.87, *P* = 0.108), while children without wasting had a median LRR of 0.33 (IQR: 0.22–0.82, *P* = 0.052). Forty (29%) hospitalized and 10 (12%) community children had LRRs above the 95th percentile value from the United States (19).

**TABLE 2.** Median lactulose rhamnose results for the children leaving hospital and their community peers across test time periods

	Hospitalized	Community
	Median (range)	Median (range)
Pre-dose	n = 33	n = 37
Rhamnose, µg/mL	0.00 (0.00–2.40)	0.00 (0.00–7.90)
Lactulose, µg/mL	0.00 (0.00–51.00)	0.00 (0.00–4.70)
Post-dose (first hour)	n = 84	n = 51
Rhamnose recovery, %*	0.10 (0.00–1.39)	0.22 (0.00–2.33)
Lactulose recovery, %	0.01 (0.00–1.37)	0.01 (0.00–1.11)
LRR	0.41 (0.02–8.33)	0.25 (0.05–4.75)
Post-dose (second hour)	n = 100	n = 72
Rhamnose recovery, %*	0.38 (0.00–5.76)	0.51 (0.00–3.31)
Lactulose recovery, %	0.02 (0.00–0.90)	0.03 (0.00–0.29)
LRR	0.30 (0.04–4.55)	0.26 (0.05–2.81)
Post-dose (cumulative)†	n = 137	n = 84
Rhamnose recovery, %	0.36 (0.00–5.76)	0.59 (0.00–4.02)
Lactulose recovery, %	0.02 (0.00–1.37)	0.03 (0.00–1.11)
LRR	0.36 (0.04–8.33)	0.27 (0.05–4.75)
Above USA 95th percentile	29%	12%

LRR = lactulose-rhamnose ratio.

* Lowest rhamnose readouts were >0, but rounded values are presented.

† Cumulative fractional rhamnose and lactulose are calculated by adding the fractional recovery from both time periods if a child passed urine in both periods, or if not the available result from only the time period urine was passed. The cumulative LRR is the mean concentration of lactulose recovered in both periods, weighted by the volume of urine recovered in that period, over a similarly weighted mean of concentration of rhamnose.

In the log-LRR models with a random effect for site, hospitalized children had mean log-LRR 0.43 [95% confidence interval (CI): 0.15–0.71, *P* = 0.003] higher than the community group. After adjustment for WLZ, the difference in log-LRR was 0.31 (95% CI: 0.00–0.62, *P* = 0.049). No other variables further confounded this association. Exclusion of children with HIV or a history of recent diarrhea did not alter the results.

### LRR and Growth Among Hospitalized Children

**FIGURE 2.**
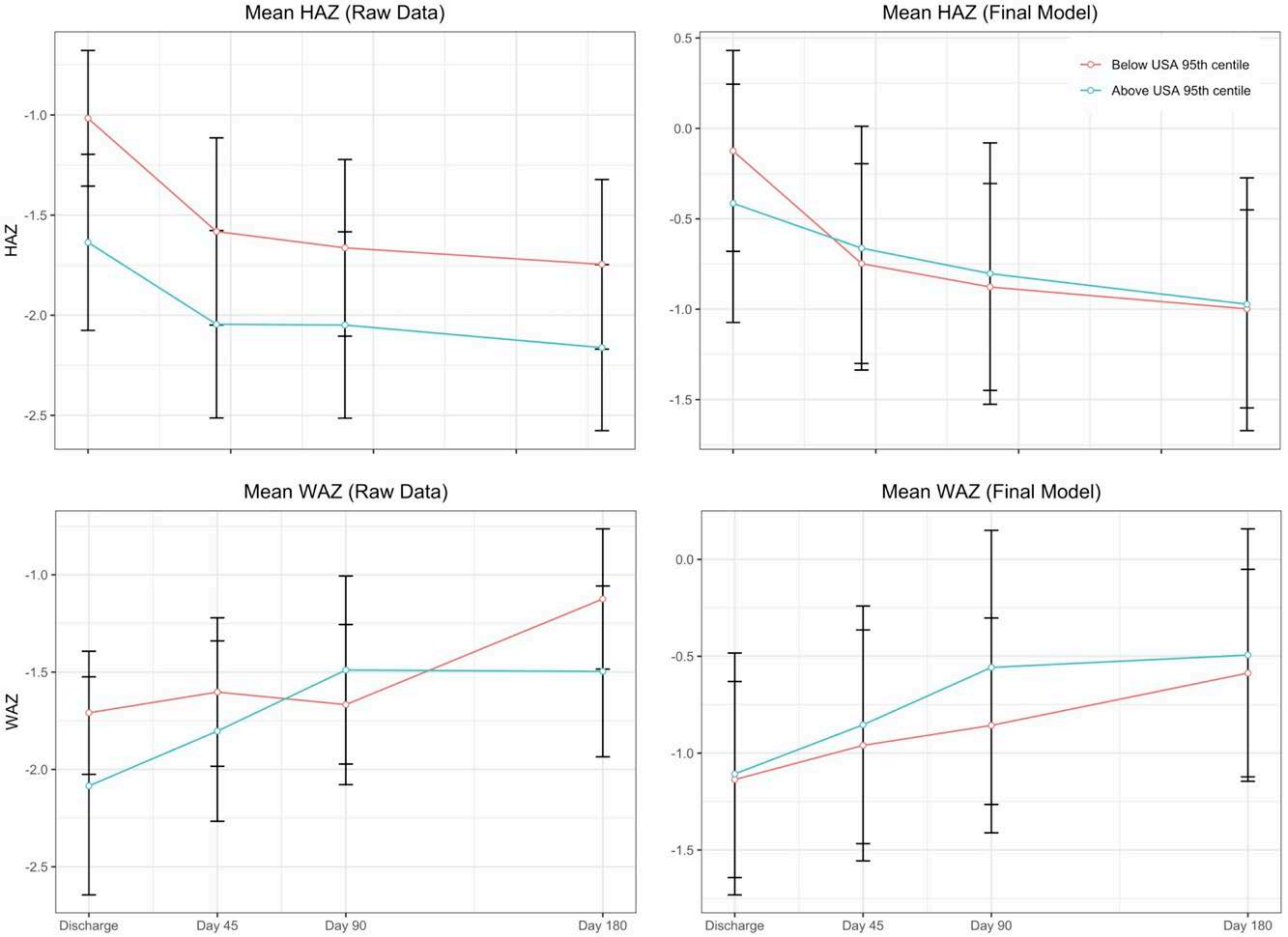
Growth patterns in the post-discharge period, stratified by lactulose-rhamnose ratio results above (green line) or below (red line) the 95th centile value for the lactulose-rhamnose ratio test in a population of similarly age children from the United States. Raw data are means and 95% confidence intervals without missing data imputation and without adjustment. The final model estimates are from a linear mixed effect model with random effect for individual, missing data imputation and adjusted for age, sex, site, baseline LAZ, baseline WAZ, and recent diarrhea. LAZ = length-for-age *z* score; WAZ = weight-for-age *z* score.

During follow-up 5 (4%) children died. LRR did not differ between children who died (median: 0.38, IQR: 0.34–0.41) and those who survived (median: 0.35, IQR 0.22–0.92, *P* = 0.805). Across 528 possible height measures for the 132 survivors, 426 (81%) were collected. There was no evidence that log-LRR was associated with ΔLAZ in crude or adjusted linear mixed effect models (Fig. 2, Supplemental Digital Content 2.3, http://links.lww.com/MPG/C941). Of 528 possible weight assessments, 427 (81%) were collected. Again, there was no association between log-LRR and ΔWAZ. The results using percentage lactulose did not differ from primary models, nor did analysis of subpopulations of children with and without recent diarrhea.

### LRR and Plasma Biomarkers

Among children selected for proteomic analysis, 91 of 111 (82%) hospitalized and 64 of 64 (100%) community children had plasma available (Supplemental Digital Content 2.4, http://links.lww.com/MPG/C941). CRP levels were 0.50 (95% CI: 0.18–0.81; *P* = 0.002) SD higher among hospitalized compared to community children. Similarly, TNFα, IL-6, and I-FABP were 0.67 (95% CI: 0.36–0.97; *P* < 0.001), 0.33 (95% CI: 0.01–0.65; *P* = 0.043), and 0.35 (95% CI: 0.04–0.67; *P* = 0.029) SD higher among the children leaving hospital, respectively. Mean CD14 levels were also elevated but not statistically significant (SD: 0.29; 95% CI: −0.03 to 0.61; *P* = 0.078, Table 3).

**TABLE 3.** The association between LRR and plasma biomarkers of systemic inflammation and intestinal damage

	Mean difference* (hospital-community)	Association with LRR*		Interaction term
		Hospitalized	Community	
	Coefficient (95% CI)	Coefficient (95% CI)	Coefficient (95% CI)	*P* value
I-FABP	0.35 (0.04–0.67)†	0.013(−0.05 to 0.30)	0.21 (−0.03 to 0.45)	0.548
CD14	0.29 (−0.03 to 0.61)	−0.02 (−0.19 to 0.15)	0.21 (−0.02 to 0.44)	0.078
CRP	0.50 (0.18–0.81)†	−0.15 (−0.32 to 0.02)	0.13 (−0.11 to 0.36)	0.036†
IL-6	0.33 (0.01–0.65)†	0.07 (−0.10 to 0.24)	0.22 (−0.01 to 0.46)	0.243
TNFα	0.67 (0.36–0.97)†	0.04 (−0.13 to 0.20)	0.34 (0.11–0.56)†	0.017†

CRP = C-reative protein; CD14 = cluster of differentiation 14; TNFα = tumour necrosis factor alpha; IL-6 = interleukin-6; I-FABP = intestinal fatty-acid binding protein; LRR = lactulose-rhamnose ratio.

* All units are standard deviations.

† *P* < 0.05.

There were interactions between log-LRR and hospital/community status for CRP (*P* = 0.036), CD14 (*P* = 0.078), and TNFα (*P* = 0.017) suggesting that log-LRR had different associations with these biomarkers in hospitalized compared to the community children. Among the community group, a 1 unit increase in log-LRR was associated with a 0.34 (95% CI: 0.11–0.56, *P* = 0.004) SD increase in TNFα but this association was not seen in the hospitalized cohort (coef: 0.04, 95% CI: −0.13 to 0.20, *P* = 0.665). Log-LRR was associated with a nonsignificant 0.21 SD (95% CI: −0.03 to 0.44, *P* = 0.078) increase in CD14 in the community cohort, but there was not an association among the hospitalized children (coef: −0.02, 95% CI: −0.19 to 0.15, *P* = 0.833). The interaction between log-LRR and IL-6 was not significant (*P* = 0.243). In the community group, a 1 unit increase in the log-LRR was associated with a nonsignificant 0.22 (95% CI: −0.01 to 0.44, *P* = 0.062) SD increase in IL-6. Again, no evidence of a log-LRR-IL-6 association was seen among the hospitalized children (coef: 0.07, 95% CI: −0.10 to 0.24, *P* = 0.406). Conversely, log-LRR was not associated with CRP in the community group (0.13 SD, 95% CI: −0.11 to 0.36, *P* = 0.286) but a higher log-LRR may have been associated with a lower CRP among hospitalized children (−0.15 SD, 95% CI: −0.32 to 0.02, *P* = 0.091).

The log-LRR and I-FABP relationship did not differ between hospitalized and community children (*P* = 0.548). Pooling community and hospitalized children suggested a nonsignificant association between higher log-LRR and elevated I-FABP (0.15 SD, 95% CI: −0.04 to 0.31, *P* = 0.056). Models using percentage lactulose replicated these findings (Supplemental Digital Content 2.5, http://links.lww.com/MPG/C941).

## DISCUSSION

Children discharged from hospital had a higher degree of enteric permeability than their community peers. The relationship between enteric permeability and systemic inflammation differed between hospitalized and community children. Among community children there was evidence that permeability was associated with systemic inflammation, as observed in other community studies (8,10,24–26). However, there was not an association between increased LRR and systemic inflammation among the hospitalized group, and we found no evidence that permeability at discharge was associated with post-discharge growth. These findings support previous studies suggesting enteric permeability is an important determinant of systemic inflammation among relatively healthy children, but our data suggest that permeability is not a predominant driver of systemic inflammation among children recovering from acute illness.

Systemic inflammation may play a key role in suppressing growth among community children, and may be caused by enteric inflammation or antigen translocation from the gut into the circulation (8,24,27). TNFα and IL-6 in particular have been implicated in the association between chronic inflammatory conditions and growth, including environmental enteric dysfunction, inflammatory bowel disease, and juvenile arthritis (28,29). These biomarkers are acute-phase proteins and, in the context of severe illness, enteropathy may not be the only driver of these cytokines. Our hospitalized group had higher biomarker concentrations than community children, presumably because they were recovering from infectious illnesses. It is likely that these recent infections are more potent stimulants of systemic inflammation than enteropathy.

The relationship between enterocyte damage and enteric permeability was similar in community and hospitalized children. Alternative mechanisms, such as malabsorption and reduced oral vaccine responsiveness (7,9), may link enterocyte damage and enteric permeability to negative outcomes independently from the systemic inflammatory pathway, suggesting enteropathy could still adversely affect child health after discharge. Additionally, LRR at discharge will not reflect enteric permeability for the entire post-discharge period. Future studies should assess if changes in permeability after hospital discharge are a determinant of systemic inflammation and growth later in the post-discharge period. Nevertheless, the lack of association between permeability and systemic inflammation at discharge, and growth in the subsequent 45 days, suggests that permeability targeted interventions may have limited impact on early post-discharge outcomes.

Our LRR values were comparable to a similarly aged Peruvian children, but lower than those from a Zambian cohort (19). We also observed associations with known correlates of enteric permeability, including wasting and systemic inflammation in the community group. Collectively, these observations indicate that the LRR performed similarly in our study to enteric permeability work conducted by other researchers. However, there was notable heterogeneity between the sites, which suggests that the generalizability of our finding to other settings may be limited.

This study’s strengths included highly standardized LRR procedures, rich medical and social phenotyping of participants, and quantitative proteomics. However, it has several limitations. The LRR is influenced by gastric emptying, intestinal motility, and the frequency of urinary voiding (13), which introduces a nondifferential misclassification into our results. Age and sex have been shown to influence dual sugar test results. While we did not use age-standardized permeability scores as used in lactulose-mannitol tests, we did adjust for age and sex in our models (24). This analysis also included a relatively small number of children, and consequently could not comment on the association between LRR and death or rehospitalization, nor could it definitively explore inter-site and inter-syndrome heterogeneity. Our sample size, and the 6-month follow-up duration, may have precluded detection of small associations with post-discharge growth. Finally, while we did minimize follow-up visit frequency, it is possible that care administered during these visits may have affected the relationship between LRR and post-discharge outcomes.

## CONCLUSIONS

Children recovering from acute illnesses had worse enteric barrier function than their community peers. However, we found no evidence to support an association between LRR at discharge and subsequent growth in the post-discharge period. We also found LRR to have different relationships with systemic inflammatory biomarkers in community and hospitalized children, with effect estimates suggesting permeability is not a driver of systemic inflammation among children at hospital discharge. These data suggest that findings of community cohorts may not be generalizable to children recovering from acute illness. Interventions aiming to reduce enteric permeability may not be effective in reducing adverse child health outcomes in the early post-discharge period.

## Supplementary Material


