# Supplementary material for: Enteric Permeability, Systemic Inflammation, and Post-Discharge Growth Among a Cohort of Hospitalized Children in Kenya and Pakistan
Source: J Pediatr Gastroenterol Nutr. 2022 Sep 20;75(6):768–74. doi: 10.1097/MPG.0000000000003619 (PMC9645542; doi:10.1097/MPG.0000000000003619)
Supplement: Supplementary file 1 [file mpg-75-0768-s001.pdf]

Supplemental digital content 1: Formulae for calculation of sugar recovery and LRR:

Lactulose or rhamnose recovery in each timepoint:

$$Sugar\ recovery\ in\ any\ timepoint = {[Sugar] * volume\ of\ urine}/{Dose\ of\ Sugar\ administered}$$

Overall:

$$Sugar\ recovery\ overall = Sugar\ recovery_{t1} + Sugar\ recovery_{t2}$$

Lactulose:Rhamnose ratio for each time period

$$LRR_{tx} = {[Lactulose]_{tx}}/{[Rhamnose]_{tx}}$$

Overall:

$$LRR_{overall} = \frac{\left([L]_{t1} \times {Vol.urine_{t1}}/{total\ urine}\right) + \left([L]_{t2} \times {Vol.urine_{t2}}/{total\ urine}\right)}{\left([R]_{t1} \times {Vol.urine_{t1}}/{total\ urine}\right) + \left([R]_{t2} \times {Vol.urine_{t2}}/{total\ urine}\right)}$$

Supplemental digital content 2: Additional results

2.1: Pre- and Post-dose Lactulose and Rhamnose:
Pre-sugar administration urinary lactulose and rhamnose concentrations (binary: detected/not detected binary) patterns were also explored and tested for association with participant group (hospitalized/community), site, age (greater/less than 6-months) and breastfeeding status (currently receiving any breastmilk/not, and exclusive breastfeed/not) using Chi-square tests.

Seventy children passed urine prior to administration of the sugars. Lactulose was detected in eight (22%) pre-dose urines from community and ten (27%) hospitalized children, although this difference was not statistically significant. Lactulose concentrations in pre-dose samples were substantially lower than in post-dose samples. The pre-dose median lactulose concentration among samples with rhamnose detected was 1.6 ug/ml, whereas this measure was 7.7 ug/ml in the first hour of post-dose collection and 18.0 ug/ml in the second. Among the 18 children with pre-dose lactulose detections, eight were considered significant contaminations (>3x the lower limit of detection), of which six were major contaminations (>10x the lower limit of detection).[18] Pre-dose urine samples from the Karachi site were significantly more likely to have lactulose detected (12/31, 39%) compared than those from Migori (6/39, 15%, p=0.03). Children over 6-months of age (p=0.004) and those not currently receiving any breast milk (p=0.006) had a significantly higher probability of lactulose detection in pre-dose samples. There was evidence that lactulose detection was less common among exclusively breastfeeding children (p=0.050).

Rhamnose was detected in pre-dose urines of six (16%) community and three (9%) of hospitalized children, but there was no association between its detection and site, age, or breastfeeding status. Rhamnose concentrations in pre-dose samples were also substantially lower than the concentrations in post-dose samples. The pre-dose median rhamnose concentration among samples with rhamnose detected was 2.4 ug/ml, whereas this measure was 22.5 ug/ml in the first hour of post-dose collection and 63.0 ug/ml in the second. Among the nine children with pre-dose rhamnose detected, eight were classified as significant contaminations (>3x the lower limit of detection), and two of these met the criteria major contamination (>10x the lower limit of detection).[18]

Among 221 children with successful tests, 135 (61%) passed urine in the first hour after sugar solution administration, and 172 (78%) passed urine in the second hour. In both the community and hospitalized groups fractional rhamnose and lactulose recovery were lower in the first hour compared to the second (Table 2). Rhamnose recovery appeared to vary widely across site and time period (supplementary table 1), with the community group having a significantly higher rhamnose recovery compared to the hospitalized group (0.59 [0.00, 3.31] vs 0.36 [0.00, 5.67], p=0.007). Conversely, the distribution of lactulose recovery was narrower across the different settings, and while there did appear to be greater lactulose recovery in community, it was not significantly different to the hospitalized population (0.03 [0.00, 1.11] vs 0.02 [0.00, 1.37], p= 0.100).

Supplemental digital content 2.2: Median LR values by site and community/hospital group

Both lactulose and rhamnose percent recovery were significantly higher in the community. However, the difference in lactulose recovery between the two groups was smaller, indicating that on average, the amount of lactulose recovered per unit rhamnose was higher among hospitalized children.

	Migori[1]				Karachi[1]			
	**Hospitalized**		**Community**		**Hospitalized**		**Community**	
	Median	(Range)	Median	(Range)	Median	(Range)	Median	(Range)
**Pre-dose**	n= 17		n=22		n= 16		n=15	
Rhamnose (ug/mL)	0.00	(0.00, 1.50)	0.00	(0.00, 7.20)	0.00	(0.00, 2.40)	0.00	(0.00, 7.90)
Lactulose (ug/mL)	0.00	(0.00, 51.00)	0.00	(0.00, 1.90)	0.00	(0.00, 10.00)	0.00	(0.00, 4.70)
**Post-dose (1st hour)**	n=59		n=31		n=25		n=20	
Rhamnose recovery (%)	0.15	(0.00, 1.39)	0.16	(0.00, 1.85)	0.08	(0.00, 0.83)	0.32	(0.00, 2.33)
Lactulose recovery (%)	0.01	(0.00, 1.37)	0.01	(0.00, 1.11)	0.01	(0.00, 0.08)	0.01	(0.00, 0.10)
LR ratio	0.38	(0.02, 6.13)	0.25	(0.05, 4.75)	0.54	(0.07, 8.33)	0.23	(0.09, 1.80)
**Post-dose (2nd hour)**	n=79		n=44		n=21		n=28	
Rhamnose recovery (%)	0.37	(0.00, 5.76)	0.56	(0.00, 3.31)	0.44	(0.03, 1.55)	0.35	(0.00, 1.37)
Lactulose recovery (%)	0.02	(0.00, 0.90)	0.03	(0.00, 0.29)	0.03	(0.00, 0.28)	0.02	(0.00, 0.14)
LR ratio	0.30	(0.04, 4.55)	0.23	(0.05, 0.56)	0.27	(0.07, 3.88)	0.36	(0.16, 2.81)
**Post-dose (Cumulative)[2]**	n=108		n=52		n=29		n=32	
Rhamnose recovery (%)	0.32	(0.00, 5.76)	0.71	(0.00, 4.02)	0.46	(0.00, 1.83)	0.40	(0.00, 2.84)
Lactulose recovery (%)	0.02	(0.00, 1.37)	0.03	(0.00, 1.11)	0.03	(0.00, 0.31)	0.03	(0.00, 0.23)
LR ratio	0.35	(0.04, 6.13)	0.24	(0.05, 4.75)	0.41	(0.07, 8.33)	0.39	(0.15, 2.81)
Above USA 95th percentile	28%	-- --	6%	-- --	34%	-- --	22%	-- --

[1]Note: in smaller n cells, such as the Karachi results in this table, the median doesn't adequately describe the distribution of values, and so it cannot be assumed that changes in median lactulose and rhamnose may not reflect the changes median LRR.

[2]Cumulative fractional rhamnose and lactulose are calculated by addition of the fractional recovery from both time periods if a child passed urine in both periods. The cumulative LRR is the mean concentration of lactulose recovered in both periods, weighted by the volume of urine recovered in that period, over a similarly weighted mean of concentration of rhamnose.

Supplemental digital content 2.3: Difference in growth (change in anthropometry) between time periods associated with a log increased in LRR score.

	Day 45		Day 90		Day 180	
	**Coefficient**	**(95% CI)**	**Coefficient**	**(95% CI)**	**Coefficient**	**(95% CI)**
**Difference in change in HAZ**						
Crude model	**0.02**	(-0.03, 0.07)	**0.01**	(-0.05, 0.07)	**0.01**	(-0.06, 0.07)
Adjusted[1]	**0.00**	(-0.02, 0.02)	**0.01**	(-0.01, 0.03)	**0.01**	(-0.01, 0.03)
No Diarrhea	**-0.02**	(-0.04, 0.01)	**0.01**	(-0.01, 0.03)	**0.00**	(-0.02, 0.02)
Diarrhea only	**-0.04**	(-0.01, 0.08)	**-0.01**	(-0.05, 0.02)	**0.02**	(-0.02, 0.06)
Percent Lactulose	**0.00**	(-0.01, 0.01)	**0.00**	(-0.01, 0.01)	**0.00**	(-0.01, 0.01)
**Difference in change in WAZ**						
Crude	**0.02**	(-0.05, 0.09)	**0.01**	(-0.06, 0.08)	**0.00**	(-0.09, 0.08)
Adjusted[1]	**0.02**	(-0.04, 0.09)	**0.02**	(-0.05, 0.08)	**0.00**	(-0.08, 0.08)
No Diarrhea	**0.01**	(-0.07, 0.09)	**0.01**	(-0.07, 0.08)	**0.00**	(-0.08, 0.09)
Diarrhea only	**0.03**	(-0.10, 0.15)	**0.04**	(-0.10, 0.18)	**0.00**	(-0.14, 0.13)
Percent Lactulose	**0.00**	(-0.04, 0.04)	**0.00**	(-0.04, 0.05)	**0.00**	(-0.04, 0.04)

[1]Adjusted for patient age in months, sex, site of recruitment, recent diarrhea history, HAZ at discharge, and WAZ at discharge.

Supplemental digital content 2.4: Characteristic of hospitalized and community children who had plasma proteomic and lactulose rhamnose results available.

	Hospital (N: 91)		Community (N: 64)	
	N	(%)	N	(%)
**CHILD**				
**Site**				
Migori	75	(82)	37	(58)
Karachi	16	(18)	27	(42)
**Age (months)**				
<6	26	(29)	16	(25)
6-12	29	(32)	19	(30)
≥12	36	(40)	29	(45)
**Sex (male)**	57	(63)	37	(58)
**Current breastfeeding**	58	(64)	49	(77)
**Current exclusive breastfeeding**	25	(27)	38	(59)
**Length of stay**				
<48 hours	13	(14)	--	--
2-5 days	36	(40)	--	--
>5days	42	(52)	--	--
**Recent abx**	88	(97)	10	(16)
**Stunted**	32	(35)	19	(30)
**Wasted**	47	(52)	7	(11)
**Admission diagnosis**				
Diarrhea	37	(41)	--	--
Malaria	19	(21)	1	(2)
LRTI	46	(51)	--	--
SIRS	29	(32)	--	--
**HIV**				
Unexposed	73	(80)	56	(88)
Expose uninfected	11	(12)	7	(11)
Infected	7	(8)	1	(2)
**Chronic illness**	7	(8)	2	(3)
**CAREGIVER**				
**Biological mother**	85	(93)	59	(92)
**Education**				
None	10	(11)	15	(24)
Primary	54	(59)	32	(52)
Secondary	27	(30)	15	(24)
**BMI**				
Underweight	10	(11)	1	(2)
Normal	67	(74)	41	(64)
Overweight	12	(13)	21	(33)
**SES**				
**Livestock owned**	51	(56)	34	(53)

**Improved water source**	64	(70)	49	(77)
**Improved sanitation**	42	(46)	35	(55)
**Food Insecurity**				
Low	31	(34)	26	(41)
Moderate	39	(43)	31	(48)
High	21	(23)	7	(11)

Supplemental digital content 2.5: The association between percentage lactulose recovery and plasma biomarkers of systemic inflammation and intestinal damage.

	Association with Lactulose Recovery		Interaction term
	Community	Hospitalized	
	Coefficient (95%CI)	Coefficient (95%CI)	P-value
**I-FABP**	0.09 (-0.04, 0.22)	0.03 (-0.08, 0.14)	0.224
**CD14**	0.14 (0.02, 0.27)*	0.07 (-0.03, 0.17)	0.136
**CRP**	0.15 (0.03, 0.28)*	0.01 (-0.09, 0.12)	0.004
**IL-6**	0.16 (0.04, 0.28)*	0.06 (-0.04, 0.16)	0.044
**TNFα**	0.21 (0.09, 0.32)*	0.07 (-0.03, 0.17)	0.004

*P <0.05
